# DeepDualEnhancer: A Dual-Feature Input DNABert Based Deep Learning Method for Enhancer Recognition

**DOI:** 10.3390/ijms252111744

**Published:** 2024-11-01

**Authors:** Tao Song, Haonan Song, Zhiyi Pan, Yuan Gao, Huanhuan Dai, Xun Wang

**Affiliations:** Qingdao Institute of Software, College of Computer Science and Technology, China University of Petroleum, Qingdao 266555, China; tsong@upc.edu.cn (T.S.); z22070004@s.upc.edu.cn (H.S.); z22070001@s.upc.edu.cn (Z.P.); z22070008@s.upc.edu.cn (Y.G.); s21070055@s.upc.edu.cn (H.D.)

**Keywords:** enhancer, deep learning, genomic signal, DNABert

## Abstract

Enhancers are cis-regulatory DNA sequences that are widely distributed throughout the genome. They can precisely regulate the expression of target genes. Since the features of enhancer segments are difficult to detect, we propose DeepDualEnhancer, a DNABert-based method using a multi-scale convolutional neural network, BiLSTM, for enhancer identification. We first designed the DeepDualEnhancer method based only on the DNA sequence input. It mainly consists of a multi-scale Convolutional Neural Network, and BiLSTM to extract features by DNABert and embedding, respectively. Meanwhile, we collected new datasets from the enhancer–promoter interaction field and designed the method DeepDualEnhancer-genomic for inputting DNA sequences and genomic signals, which consists of the transformer sequence attention. Extensive comparisons of our method with 20 other excellent methods through 5-fold cross validation, ablation experiments, and an independent test demonstrated that DeepDualEnhancer achieves the best performance. It is also found that the inclusion of genomic signals helps the enhancer recognition task to be performed better.

## 1. Introduction

Defining transcriptional regulatory signals is crucial for understanding eukaryotic gene expression [1]. In bioinformatics, enhancers and super-enhancers are positive regulators of gene transcription [2]. There is substantial evidence that enhancer-based transcriptional regulation is involved in determining cell fate and tissue development [3,4,5]. For example, the mammalian chromosome architecture is thought to regulate transcription by modulating three-dimensional interactions between enhancers and promoters [5,6,7]. Large-scale sequencing studies have identified a large number of genetic variants in enhancers that regulate genes through remote chromatin interactions [8,9]. At the same time, the role of enhancers in cancer development is receiving increasing attention [8,10]. Research on enhancers will make an important contribution to disease progression.

Current methods for identifying enhancers are categorized into two main groups: high-throughput experiments and computational methods. High-throughput experiments include chromatin immunoprecipitation followed by deep sequencing (ChIP-seq) [11,12], protein-binding microarrays (PBMs) [13], and the systematic evolution of ligands by exponential enrichment (SELEX) [14]. The most common method is ChIP sequencing (ChIP-seq). It combines chromatin immunoprecipitation (ChIP) and massively parallel sequencing technologies to identify mammalian DNA sequences bound by transcription factors in vivo.

In the past, high-throughput experiments have made a great contribution to enhancer identification. But they have two drawbacks that are hard to overcome: they take a lot of time and use a lot of resources. In recent years, machine learning has reformulated the enhancer identification problem as a binary classification task to predict how enhancer regions differ from non-enhancer regions (negative samples) [15]. Machine learning methods such as Support Vector Machines (SVMs), Artificial Neural Networks (ANNs), Random Forests (RFs), Probabilistic Graphical Models (PGMs), and integration techniques have been successfully applied in the field of enhancer prediction with good experimental results. Probabilistic graphical models such as ChromHMM [16] use a probabilistic model based on multivariate HMM. ChromHMM partitions the genome into 200 bp intervals and trains a single model on data from six available cell lines. SVM is a machine learning method that is widely applicable to many types of pattern recognition problems. ChromaGenSVM [17] systematically trains SVMs with the chromatin epigenetic marks associated with enhancers. It is the first SVM model to discover functionally regulated regions from histone methylation maps. The random forest model iEnhancer-RF [18], with enhanced feature representation, predicted enhancers and their strengths in the genome. Better results were achieved compared to most of the methods available at that time.

With the development of deep learning techniques in recent years, many complex problems in bioinformatics have been solved by deep learning tools. In the field of enhancer recognition, many deep learning methods are proposed and achieve good prediction performance. For example, iEnhancer-GAN [19] employs Seq-GAN to generate non-enhancers, strong enhancers, and weak enhancers for a relatively small training dataset, while a CNN architecture is designed to integrate feature extraction and recognition tasks. iEnhancer-RD [20] proposes the new feature methods KPCV and RKPK, and compares these two representations. iEnhancer-RD uses Deep Neural Networks (DNNs) as classifiers to significantly improve the performance of the prediction tool. As the LSTM (Long Short-Term Memory Network) has been proposed and widely used in various fields, iEnhancer-EBLSTM [21] utilizes the advantages of LSTM and establishes a bi-directional LSTM network-based enhancer recognition method. BERT is used as a common encoding method to better represent the input features. Le et al. [22] found that deep learning has the greater potential to learn BERT features than other traditional machine learning techniques. A method based on BERT and a 2D convolutional neural network was implemented to recognize DNA enhancers from sequence information.

There have been many deep learning methods applied to the field of enhancer recognition. Superior prediction results have been realized, but there are still some problems. The first is that most of the encoding approaches used for the input sequences are One-Hot, and do not express the sequence features properly. Meanwhile, the existing network architectures are not good at extracting effective shallow and deep features. Finally, inputting only the DNA sequence does not necessarily achieve the best prediction results. In order to solve the above problems, we propose DeepDualEnhancer to complete the enhancer recognition task:

1. The two encoding approaches of fine-tuned DNABert and embedding are used to convert the enhancer sequences into feature matrices, which can better represent different sequence feature information.
2. A two-channel network architecture is designed to process the two feature matrices. We use a combination of multi-scale CNN and BiLSTM to better extract shallow and deep features; this is so that our model achieves the best prediction effect among other existing methods.
3. Enhancer datasets including six different cell lines were collected from the enhancer–promoter interaction field. The datasets were pre-processed with operations such as redundancy removal. Meanwhile, we designed a network architecture based on genomic signals and a DNA sequence. The network architecture was designed using the transformer sequence attention. The network architecture incorporating genomic features was compared with other methods on a new dataset and optimal results were achieved.

## 2. Results

### 2.1. Reveal TF Motifs Required for Enhancers

**Figure 1:**
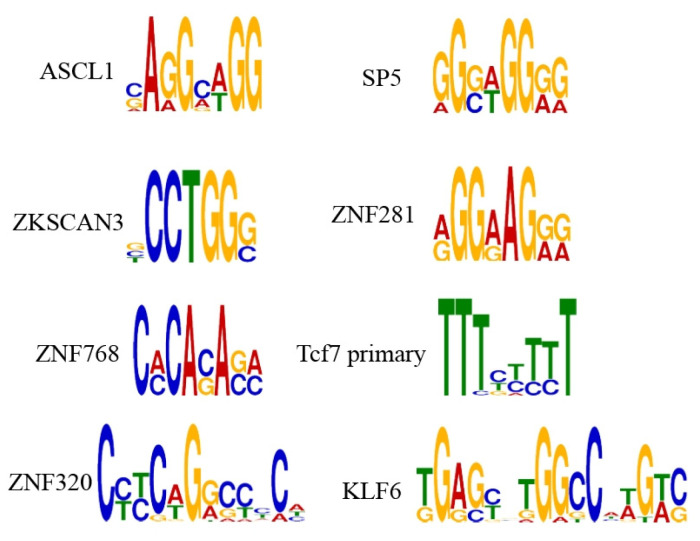
DeepDualEnhancer motifs analyzed by MEME suite.

In both the training set and the independent test set, we found a large number of identical known motifs in the correctly predicted enhancement subsequences of DeepDualEnhancer; these were the DeepDualEnhancer motifs, which were generated by MEME suite by summarizing the pattern of repetitively predicted sequences for all correctly predicted enhancement subsequences. The results demonstrate that our model finds many motifs in unknown DNA sequences, proving the strong learning ability of our model. We selected some motifs found by the model, as shown in Figure 1.

### 2.2. Cross Validation

To test the predictive performance of the DeepDualEnhancer, we used 5-fold cross-validation. In 5-fold cross-validation, the training dataset is divided into five equal or approximately equal sized parts, four of which are used to train the model and the rest are used to test the model. This process was repeated five times. Many previous methods of enhancer recognition have used cross-validation to train their models. We compared the performance of our model with 12 excellent prediction methods during cross-validation on stage 1 and stage 2 tasks. In the stage 1 task. Our model outperforms most of the models on the ACC, MCC, and AUC metrics, including the recent model Enhancer-LSTMAtt. There is also a large improvement. In the stage 2 task, our model also outperforms most models. However, there is a difference between it and individual methods such as EnhancerP-2L. And the ACC and other metrics of most deep learning models decline compared to the first stage.

Deep learning models usually have more parameters and stronger expressive power. In cross-validation, due to data partitioning, the model may not generalize well. The task of dividing strong enhancers and weak enhancers in the second stage is more complex, resulting in a poorer performance. However, our model achieves the best performance on the later independent test set. The results are shown in Table 1 and Table 2.

### 2.3. Compared with State-of-the-Art Methods

**Figure 2:**
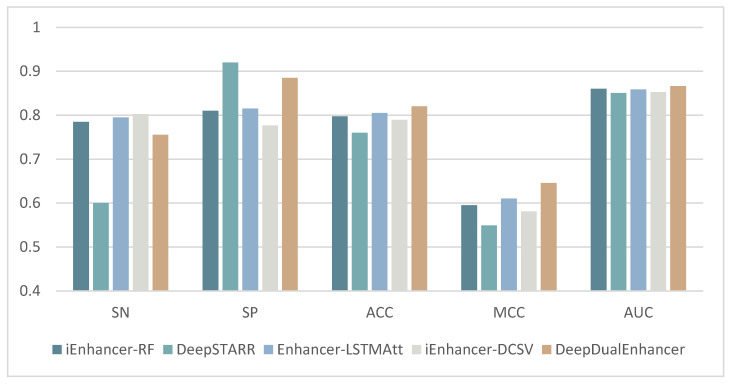
Comparison of DeepDualEnhancer with four selected well-performing methods (Stage 1).

**Figure 3:**
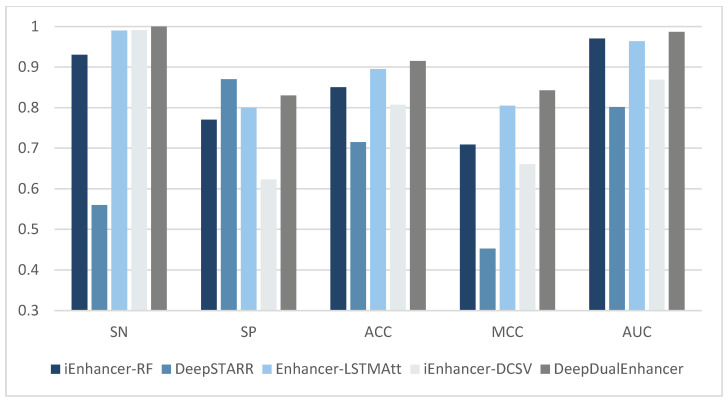
Comparison of DeepDualEnhancer with four selected well-performing methods (Stage 2).

The previous enhancer recognition methods have basically been trained and independently tested on the same dataset. Therefore, in this section, we compare DeepDualEnhancer with the past 21 enhancer recognition methods. The results are shown in detail in Table 3 and Table 4 below. Also, for a more direct comparison, we selected the four methods that performed better on the independent test set and compared them with DeepDualEnhancer on the bar chart shown in Figure 2 and Figure 3. Firstly, in stage 1 of the enhancer recognition task, our model takes the lead in most of the metrics. Our method achieves 0.8850 on the Specificity (SP) metric, which is better than 21 other machine learning methods and deep learning methods. In the accuracy (ACC) metric, our model achieves the best value of 0.8200, which is 1.5% better than the recent deep learning method Enhancer-LSTMAtt, and 3.05% better than the iEnhancer-DCSV method based on DenseNet with an attention module, and 2.25% better than the machine learning method iEnhancer-RF. On the MCC metric, our model achieves a large improvement (0.6455), which is 3.54% better than the second-best Enhancer-LSTMAtt method (0.6101) and 3.56% better than the third-best piEnPred method (0.6099). On the very important AUC metric, our model similarly achieves the best AUC value (0.8662) among the 21 methods. Compared to most of the methods, our model’s performance is much improved. We achieved a 1.35% improvement over the most recent method, iEnhancer-DCSV (0.8527), a 0.62% improvement over the second-best random forest method, iEnhancer-RF (0.8600), and a 1.56% improvement over DeepSTARR [23]. Although our model did not achieve a large improvement in the AUC metric relative to individual models, we did achieve multiple metrics at the same time; for example, we also achieved a large improvement in the MCC. Thus, our model is able to better cope with unbalanced data in real situations. It is demonstrated that our model can accomplish the enhancer recognition task excellently while outperforming other current methods in performance.

In the second stage of the strong and weak enhancer recognition task, our method achieves the best performance among all compared methods on the SN metric. It achieves the third-best value of 0.8300 on the SP metric, third to DeepSTARR’s 0.8700 and Enhancer-DRRNN’s 0.8400. Meanwhile, our method achieves an accuracy of 0.9150, which is a 2% improvement over the second-best Enhancer-LSTMAtt method (0.8950), having a large increase in accuracy. The improvement in the MCC metric is even more significant. Our method achieves an MCC value of 0.8423, which is a 3.76% improvement over the second-best Enhancer-LSTMAtt deep learning method (0.8047) and a 13.32% improvement over the machine learning method iEnhancer-RF (0.7091). It also improves by 18.14% compared to the most recent method, iEnhancer-DCSV (0.6609). Among the 20 methods compared, our model achieves the highest AUC value (0.9864), which is a 1.64% improvement over the second-best iEnhancer-RF method (0.9700). It is demonstrated that our method performs well in the task of recognizing strong and weak enhancers.

### 2.4. Ablation Experiments

For the feature matrix obtained from DNABert, we perform the further extraction of features by multiscale CNN. The combination of different convolutional kernels in multiscale CNN is crucial to the results.

In the first stage, the convolutional kernel combination 1/3/5 achieved the best SP value (0.9100) and the convolutional kernel combination 5/7/9 achieved the best SN value (0.7700). But overall, the convolutional kernel combination 3/5/7 performed the best. Convolutional kernel combination 3/5/7 achieved the best ACC (0.8200), which is about a 2% improvement over the other combinations. It also achieved the best MCC value (0.6455), a 2.6% improvement over the second-best convolutional kernel combination 1/3/5 (0.6195). Convolutional kernel combination 3/5/7 achieved an AUC of 0.8662 and also outperformed all other convolutional kernel combinations. Thus, in the first stage, the convolutional kernel combination 3/5/7 was the best performer. In the second stage of the task, the convolutional kernel combination 3/5/7 achieved values of 1.0000, 0.8300, 0.9150, 0.8423, and 0.9864 on the SN, SP, ACC, MCC, and AUC metrics, respectively. Except for AUC, the other four metrics achieved the best results among all combinations. The performance on AUC values also achieved better results.

**Figure 4:**
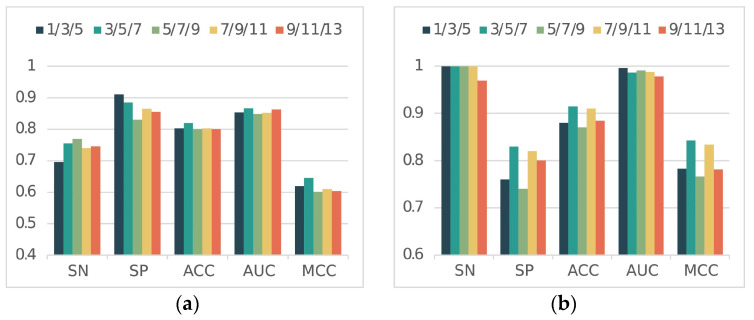
Results of ablation experiments. (**a**) and (**b**) indicate the comparison of experimental results at Stage 1 and Stage 2, respectively.

Therefore, the convolutional kernel combination 3/5/7 was able to make our method perform better in both the first and second-stage tasks. A comparison of the results for different convolution kernels is shown in Figure 4 below.

### 2.5. Compared with State-of-the-Art Methods on a New Dataset

**Figure 5:**
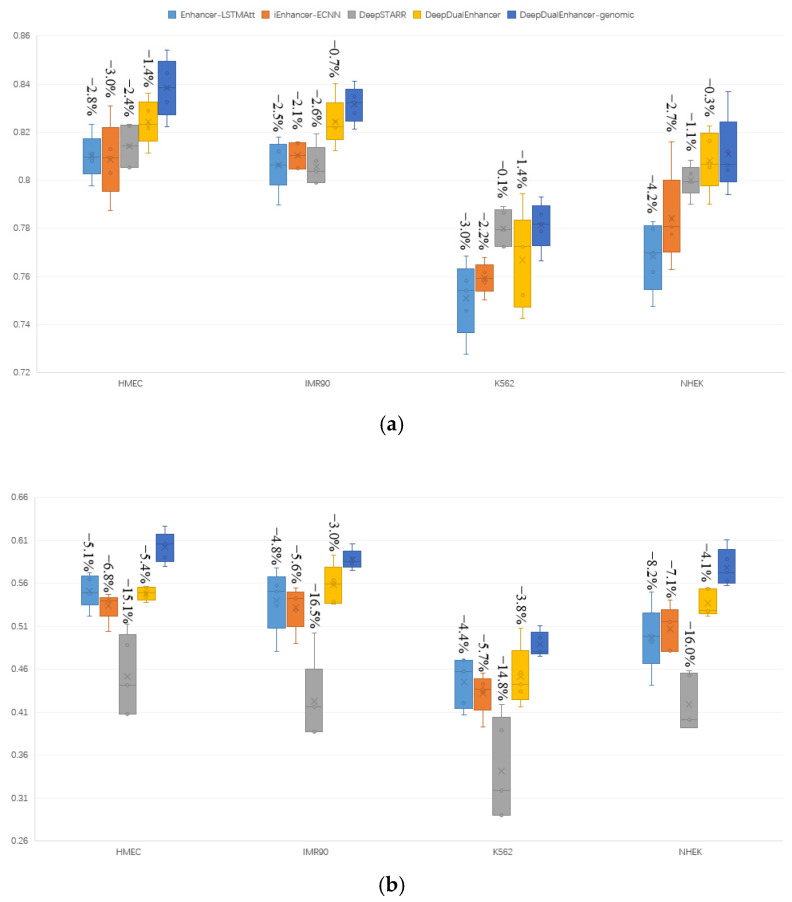

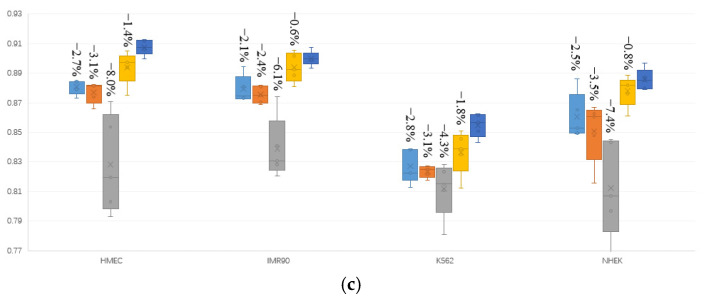
Comparison results on new datasets. (**a**) ACC metrics. (**b**) MCC metrics. (**c**) AUC metrics.

Figure 5 shows the comparison of the ACC, MCC, and AUC scores for different methods on the new imbalanced dataset. We labeled the gap values for each method and DeepDualEnhancer-genomic. The box plot results were obtained through several experiments. Since most of the past excellent methods are not open source, we selected three methods with open source code, namely Enhancer-LSTMAtt, iEnhancer-ECNN, and DeepSTARR, for comparison experiments.

Figure 5a represents the ACC metrics scores of the different methods on the four datasets. The method with the best performance on the HMEC dataset is DeepDualEnhancer-genomic (0.8384), with a large improvement compared to the other methods. This was followed by DeepDualEnhancer (0.8231), DeepSTARR (0.8144), Enhancer-LSTMAtt (0.8097), and iEnhancer-ECNN (0.8093). On the IMR90 dataset, the Enhancer-LSTMAtt (0.8063) and DeepSTARR (0.8037) scores were almost identical, and DeepDualEnhancer-genomic (0.8323) achieved the best performance. On the K562 dataset, DeepSTARR (0.7794) and DeepDualEnhancer-genomic (0.7819) performed the best. DeepDualEnhancer-genomic (0.8065) on NHEK achieved the best ACC.

A comparison of the MCC scores is shown in Figure 5b. Due to being tested on an imbalanced dataset, the MCC score of the model on an independent test set is crucial. DeepDualEnhancer-genomic performed the best on all four datasets, namely HMEC (0.6064), IMR90 (0.5858), K562 (0.4811), and NHEK (0.5729). In addition, DeepDualEnhancer and Enhancer-LSTMAtt achieved the second-best test performance on four cell lines. All are better than iEnhancer-ECNN and DeepSTARR.

Figure 5c represents a box plot comparison of the AUC scores. On all four datasets, it was DeepDualEnhancer-genomic that performed the best. On the HMEC dataset, it was DeepDualEnhancer-genomic (0.9076), DeepDualEnhancer (0.8973), Enhancer-LSTMAtt (0.8843), iEnhancer-ECNN (0.8815), and DeepSTARR (0.8197), in that order. On the IMR90 cell line, the scores were 0.8999 (DeepDualEnhancer-genomic), 0.8926 (DeepDualEnhancer), 0.8751 (iEnhancer-ECNN), 0.8745 (Enhancer-LSTMAtt), and 0.8307 (DeepSTARR), in that order. On the K562 cell line and the NHEK cell line, the poorer performers were iEnhancer-ECNN and DeepSTARR.

Thus, by analyzing the three metrics on four cell lines, DeepDualEnhancer-genomic performed the best, followed by DeepDualEnhancer without genomic signal input. This proves that our method can perform prediction on different datasets as well. It also demonstrates that the inclusion of genomic signals in the enhancer identification task will help to perform the prediction task better. This section was trained and independently tested on an imbalanced dataset, which is more in line with the real-world data distribution. Our method also achieved good performance on imbalanced datasets. The experimental results show that inputting genomic signals can enable the network to learn more common features and improve the predictive performance of the method on other cell lines.

### 2.6. Visualization of Feature Dimensionality Reduction Based on t-SNE

**Figure 6:**
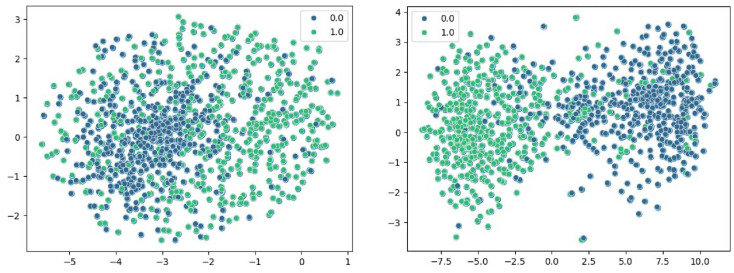
t-SNE classification results for embedding (**left**) and DNABert (**right**). Here, 0.0 and 1.0 represent label classification, and the x-coordinate and y-coordinate represent the distance after dimensionality reduction.

As shown in Figure 6, T-SNE (t-distributed Stochastic Neighbor Embedding) is a dimensionality reduction algorithm commonly used to map high-dimensional data into two or three dimensions for visualization and analysis. For the embedding layer, we used a pre-trained model to transform the DNA sequences into embedding vectors to obtain a feature matrix. For the DNABert approach, we used the pre-trained and fine-tuned DNABert to encode the gene sequences to obtain another feature matrix. These two feature matrices represent the feature expression of DNA sequences under different encoding approaches, respectively.

Next, we input these two feature matrices into the t-SNE algorithm for dimensionality reduction and visualization, respectively. The experimental results show that the sequence features obtained by DNABert show a tendency to be clearer and more clearly categorized after t-SNE dimensionality reduction. This implies that DNABert is able to better capture the similarities and differences between DNA sequences, making it easier for DNA sequences with similar functions or structures to be clustered together. In this figure, 0.0 and 1.0 represent non-enhancer samples and enhancer samples, respectively. The units of the *x*-axis and *y*-axis have no special meaning and represent the feature coordinates of the reduced sample.

### 2.7. Ablation Experiments for Genomic Signals

**Figure 7:**
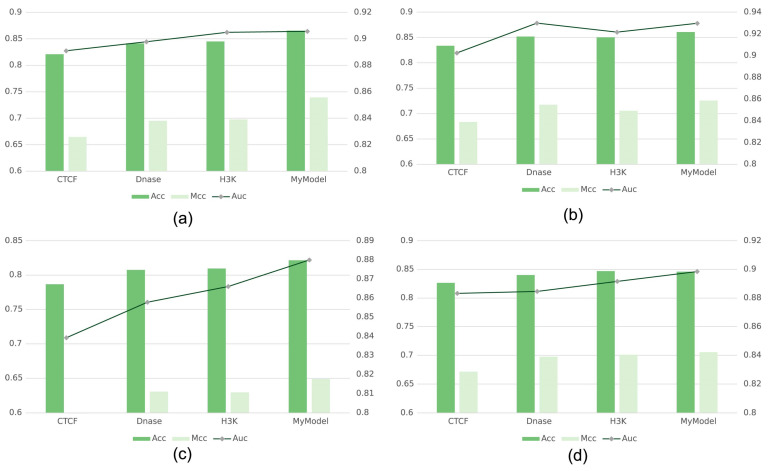
Results of genomic characterization ablation experiments on balanced BENGI dataset: (**a**) HMEC cell line; (**b**) IMR90 cell line; (**c**) K562 cell line; (**d**) NHEK cell line.

To verify the contribution of individual genomic signals to enhancer recognition, we performed ablation experiments based on genomic signals on the balanced dataset. Figure 7 shows the ACC, MCC, and AUC results on the four independent test sets of HMEC, IMR90, K562, and NHEK. First, on the HMEC cell line, the ACC score of the method lacking the CTCF signal feature was 0.8210, which was 4.44% lower than the ACC score of our method (0.8654). Also, the score on the MCC metric was unsatisfactory, at 0.6646. This is a 7.45% reduction compared to the MCC score of our method (0.7391). The change in the performance of the model missing the CTCF signal feature in AUC was not very significant, with a score of 0.8909. This is a reduction of 1.48% compared to the model with all genomic signals input (0.9057). The method with missing DNase-I features and missing histone features performed better than the method with missing CTCF features. The ACC metrics reached 0.8413 and 0.8450, respectively. The MCC metrics reached 0.6952 and 0.6978, respectively. The AUC metrics reached 0.8978 and 0.9049, respectively, which is less different from the performance of the method that inputs all the genomic features on the AUC.

The results on the IMR90 cell line are shown in Figure 7b. On the ACC metric, the scores of the methods missing CTCF, DNase-I, and histone features were 0.8336, 0.8521, and 0.8504, respectively. This is 2.73%, 0.88%, and 1.05% lower than that of the method that inputs all features (0.8609), respectively. On the MCC metric, the scores of the three methods with missing features were 0.6835, 0.7173, and 0.7055, a decrease of 4.21%, 0.83%, and 2.01% compared to the method with all genomic signals input (0.7256). On the AUC metric, the results of the ablation experiments were 0.9025, 0.9299, and 0.9216, respectively. Meanwhile, the AUC score for the missing DNase-I feature was the same as the score for the model with all genomic signals input (0.9297).

The results of the test set of K562 are shown in Figure 7c. For the ACC, MCC, and AUC, the scores of the method with missing CTCF features were 0.7868, 0.5974, and 0.8392. The scores of the method with missing DNase-I features were 0.8074, 0.6307, and 0.8578. The scores of the method with missing histone features were 0.8095, 0.6296, and 0.8660. It can be seen that the performance of the method with a missing DNase-I signal and the method with a missing histone signal are almost the same, and both of them are different from the method with all genomic signals. The methods missing the histone signal and the DNase-I signal perform almost the same. Both are not very different from the methods that input all signals. However, the scores of the method lacking CTCF signal input were 3.47%, 5.14%, and 4.07% lower than the model (0.8215, 0.6488, and 0.8799) for the three ACC, MCC, and AUC metrics, respectively.

The results for the NHEK cell lines are shown in Figure 7d. The ACC, MCC, and AUC scores of the methods lacking CTCF signal were 0.8265, 0.6713, and 0.8832, respectively. The scores of the methods lacking a DNase-I signal were 0.8401, 0.6975, and 0.8846. The scores of the methods lacking a histone signal were 0.8469, 0.7011, and 0.8916. Compared with the NHEK data set, the lack of a CTCF signal also had the greatest impact on the evaluation of the model metrics.

The above experimental results demonstrate that CTCF, DNase-I, histone, and other signals contribute to the enhancer prediction task. Each of the three input features represents different information, and the three features can complement each other’s information, which can help the model to complete the prediction more accurately. The CTCF signal had the most important contribution to the enhancer prediction task. In contrast to DNase-I and histone features, CTCF is an important transcription factor involved in the formation of the three-dimensional structure of the genome. It not only plays an important role in the interaction between enhancers and promoters, but also plays a key role in the regulation of gene expression. Compared to other signals, CTCF-binding sites may directly indicate the presence and function of enhancers, and thus the lack of CTCF signaling significantly reduces the accuracy of the model.

### 2.8. Experiments on Separate Chromosomes

**Figure 8:**
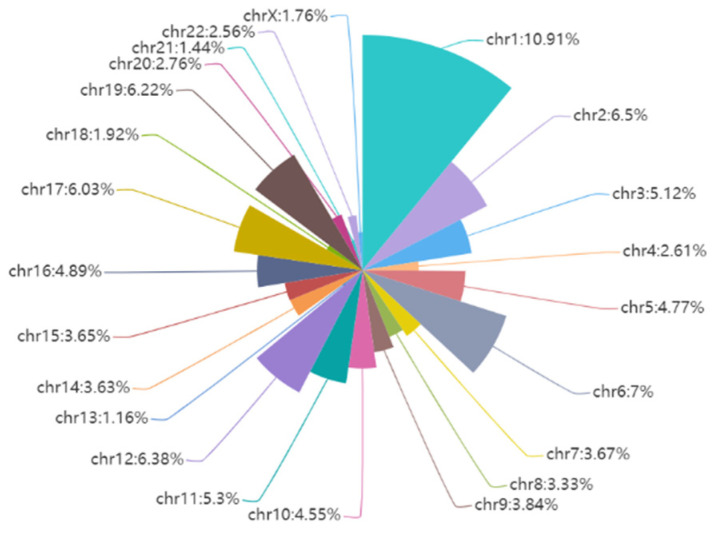
The percentage of different chromosomes on the BENGI Dataset.

**Figure 9:**
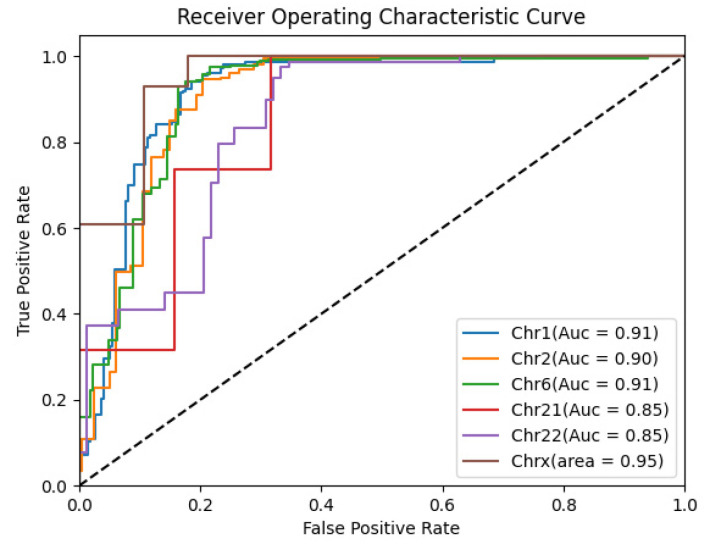
The results of the ROC curve for the independent chromosome test set.

Figure 8 shows the percentage of different chromosome species in the training set. As can be seen from the figure, there are more training data on chromosomes 1, 2, and 6, which account for a larger proportion. Meanwhile, on chromosomes 21, 22, and chromosome X, there are few training data. In order to verify that our method can perform accurate enhancer recognition tasks even on chromosomes with fewer training samples, we took out the enhancer samples on specific chromosomes in the independent test set and performed test validation. The ROC curves of the prediction results are shown in Figure 9, from which it can be seen that the AUC scores of our method for chromosomes 1, 2, and 6 were 0.91, 0.90, and 0.91, respectively. This shows that the performance is more stable. Meanwhile, the AUC scores on chromosomes 21, 22, and X were 0.85, 0.85, and 0.95, respectively. Although these scores are not as good as those on chromosomes with more training samples on average, the model still accomplishes excellent prediction results with fewer data. This proves that our model can still learn the critical features and accomplish accurate prediction with fewer training samples.

## 3. Discussion

The importance and significance of recognizing enhancer regions are great in the field of genomics and biological research. Enhancers are a class of DNA sequence regions located in the genome that play a key role in regulating gene expression. Enhancers can interact with gene promoters and other regulatory elements to regulate the level of transcription of specific genes. The method designed in this study can help medical personnel and others to better understand gene regulation and explain gene expression variation. Meanwhile, recognizing enhancer regions can help discover potential therapeutic targets and facilitate drug development and disease treatment. Our method adopts the encoding approach of DNABert, which can better capture the semantic information of DNA sequences and reduce the demand for training data through pre-training. Second, we design a dual-channel network architecture, combining a multi-scale CNN and LSTM network, that is able to extract feature information at different scales. Third, we collect imbalanced enhancer datasets from six different cell lines from other domains. Meanwhile, we design a network architecture that includes a transformer and sequence attention and that incorporates genomic signals. The method of inputting genomic signals achieves better prediction results than other state-of-the-art methods. Ultimately, it is demonstrated through multiple experiments that our method outperforms other current state-of-the-art methods. We found that better enhancer region prediction can be achieved by inputting genomic signals and DNA sequences.

Although our method achieves the best performance among existing methods, the prediction performance on some imbalanced datasets did not reach its optimal level, and the MCC score was relatively low. In the future, with the continuous development of deep learning algorithms and the improvement of model architecture, we will achieve the more accurate prediction of enhancer regions through better model design, larger-scale training data, and more effective training algorithms. At the same time, we will try to combine more multi-omics data for prediction. By combining multiple data, we can capture the features and contextual information of enhancers more comprehensively and improve the accuracy and interpretability of the prediction. We hope to further explore some features of motifs and make more detailed classification predictions for enhancers in the future. It also will further reveal the mode and mechanism of enhancers in gene regulation.

## 4. Material and Methods

### 4.1. Datasets

This study utilizes the same dataset as most previous methods. The benchmark dataset was first constructed by Liu et al. [24], based on chromatin status information from nine cell lines. The chromatin state information of the dataset was annotated by ChromHMM, a genome-wide map with multiple histone tags [16,25]. In this dataset, all samples were cut into 200 bp segments, in order to match the length of the nucleosome and linker DNA. Samples less than 200 bp in length were removed. Meanwhile, in order to prevent overfitting and remove redundant data, we used the CD-HIT tool (version 4.8.1 and threshold set to 80%) to remove DNA segments with high sequence similarity in the dataset. We used CD-HIT to reduce or eliminate homology between sequences. CD-HIT is a clustering tool used to reduce redundant sequences. Firstly, all sequence lengths were sorted, starting from the first sequence, to form a sequence class. Then, the sequences were processed sequentially. If the similarity between the next sequence and the represented sequence was too high, it was added to the same class. Otherwise, a new class was added. The generated non-redundant sequence was used to check the method’s dependence on homology. In order for the model to better recognize positive and negative samples, Chen et al. [26] selected 1484 enhancer samples and 1484 non-enhancer samples. Meanwhile, the 1484 enhancer samples were composed of 742 strong enhancer samples and 742 weak enhancer samples. In the task of distinguishing strong enhancers and weak enhancers, strong enhancers were considered positive samples while weak enhancers were considered negative samples. Also, the independent test set consisted of 100 strong enhancer samples, 100 weak enhancer samples, and 100 non-enhancer samples. The samples in the independent dataset did not appear in the training dataset. The independent test set was also processed by CD-HIT. The sequence identity between any two DNA segments did not exceed 0.8. The details of the training set and the independent test set are shown in Table 5 and Table 6.

To verify whether our method could perform well on imbalanced datasets with larger data scales, we performed the following. In the field of enhancer–promoter interactions, genomic signals are often used as inputs to predict enhancer–promoter interactions through network architectures [26,27]. We therefore processed the BENGI dataset in the enhancer–promoter interaction field to construct a new dataset. For six cell lines (GM12878, HeLa-S3, HMEC, IMR90, K562, NHEK), we extracted the enhancer segments in the EPI information as positive samples. At the same time, in order to better simulate the situation in the real world, negative samples were collected in a positive-to-negative ratio of 1:3. The details of this imbalanced large-scale dataset are shown in Table 7. GM12878 and HeLa-S3 data were used for training, and the remaining four cell lines were used for testing. The independent cell line test set could better verify the performance of the model.

**Figure 10:**
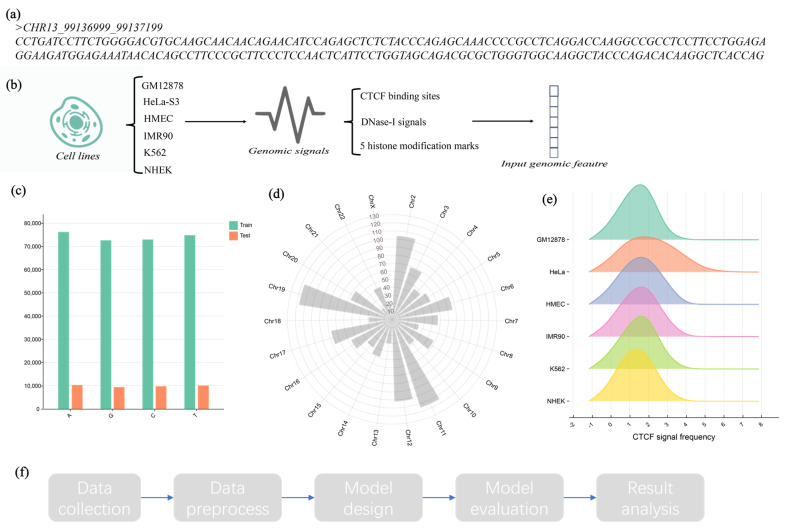
A snapshot of the dataset. (**a**) A sample of the input DNA sequence. (**b**) Introduction to genomic signals used in the new dataset. (**c**) Base distribution in the dataset. (**d**) The distribution of samples on different chromosomes in the new dataset. (**e**) Distribution of CTCF signal frequency on six cell lines. (**f**) The flowchart of this study.

Inspired by past deep learning methods based on genomic signals, we wanted to gain insight into whether genomic signals are helpful for our task. Therefore, in our work, we designed DeepDualEnhancer-genomic, which inputs genomic signals and DNA sequences in order to verify that genomic signals are helpful for the task of recognizing enhancers. The genomic and epigenomic signals included CTCF binding sites, chromatin accessibility (DNase-I signals), and 5 histone modification marks (H3K4me1, H3K4me3, H3K27me3, H3K36me3, and H3K9me3). A snapshot of the data is shown in Figure 10.

### 4.2. Network Architecture of DeepDualEnhancer

**Figure 11:**
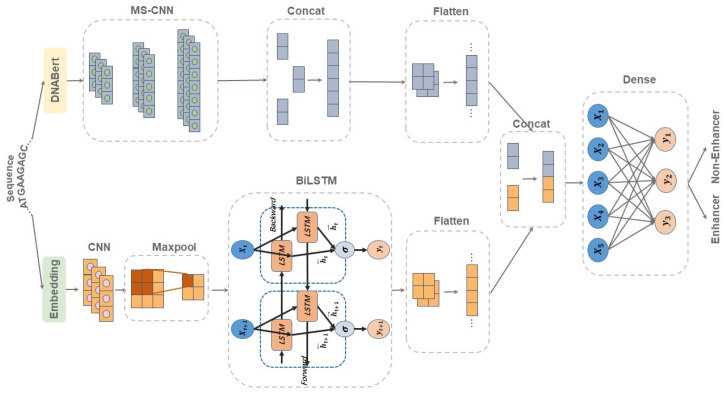
Network architecture of DeepDualEnhancer.

The input to the model is a DNA segment with a length of 200 bp. Compared to many deep learning methods in the field of enhancer recognition, two sequence encoding approaches embedding DNABert were chosen for this study. In the field of bioinformatics, embedding is often used to process sequence input. For an input DNA sequence, the embedding approach can assign a vector representation of each nucleobase. These vector representations can capture certain local features and properties of the nucleobases or nucleobase pairs. However, the embedding approach mainly focuses on local features and cannot directly capture the global information of the whole DNA sequence. Therefore, the dependencies between different nucleobases in the input sequence may not be captured well. DNABERT is a novel pre-trained bidirectional encoder representation. It captures a global and migratable understanding of genomic DNA sequences based on the nucleotide context. Therefore, our method, DeepDualEnhancer, is divided into two parts: a local feature channel containing the embedding method and a global feature channel containing DNABert. Eventually, the matrices of the two channels are subjected to concatenate operation, and the final enhancer prediction results are output through the fully connected layer. Details of the network architecture are shown in Figure 11.

#### 4.2.1. Local Feature Channel

The input DNA sequence is first converted into a continuous vector representation through an embedding layer. Embedding is a technique commonly used in machine learning and natural language processing to convert discrete data into continuous vector representation. It plays an important role in feature extraction. Compared with other traditional feature extraction methods in the field of bioinformatics, such as one-hot coding, embedding can represent nucleobases with continuous vectors. At the same time, it can capture some hidden information in the sequence and provide more abundant feature representations, which is conducive to the prediction of the model. Meanwhile, the embedding method can improve the generalization ability of the model. The embedding dimension parameter is set to 32, and the input sequence is converted into a 200 × 32 embedding matrix.

The next step is inputting the embedding matrix into a one-dimensional convolutional network. Also, after the convolutional neural network, the regularization operation of batch normalization is incorporated. The use of a batch normalization operation can improve the training speed and generalization ability of the model. In order to reduce the number of parameters and computation of our method, the feature matrix is input into the max pooling layer for a down-sample. The max pooling layer is able to extract the significant features of the input data and is robust to small changes in the input data. Bidirectional Long Short-Term Memory (BiLSTM) is used to process sequence data. The feature matrix output from the max pooling layer is fed into BiLSTM to capture the contextual information of the sequence and capture the long-term dependencies. The formula for LSTM is shown below:

In the self-attention mechanism, the input sequence is first subjected to feature extraction. It usually uses a linear transformation to map the input sequence into the Query, Key, and Value vector spaces. Specifically, for the input sequence X = {x1, x2, …, xn}, Query, Key, and Value can be generated in the following ways:

(1) $$f_{t}=\sigma \left(W_{f}\cdot \left[h_{t-1},x_{t}\right]+b_{f}\right)$$

(2) $$i_{t}=\sigma \left(W_{i}\cdot \left[h_{t-1},x_{t}\right]+b_{i}\right)$$

(3) $${\tilde{C}}_{t}=tanh\left(W_{C}\cdot \left[h_{t-1},x_{t}\right]+b_{C}\right)$$

(4) $$C_{t}=f_{t}*C_{t-1}+i_{t}*{\tilde{C}}_{t}$$

(5) $$o_{t}=\sigma \left(W_{o}\cdot \left[h_{t-1},x_{t}\right]+b_{o}\right)$$

(6) $$h_{t}=o_{t}*tanh\left(C_{t}\right)$$

where $h_{t}$ represents the hidden state; $h_{t-1}$ represents the hidden state of the last moment;$\;b_{f}$, $b_{i}$, $b_{C}$ and $b_{o}$ represent bias parameters in different gates; *W* represents the relevant weight matrices in the calculation of different gates; and $x_{t}$ represents the input at a certain moment. The value of the memory gate is $i_{t}$, and the value of the forgetting gate is $f_{t}$. The temporary cell state is denoted as ${\tilde{C}}_{t}$. The cell state is $C_{t}$, and the value of the output gate is $o_{t}$. BiLSTM obtains the hidden states $h_{t}^{f}$ and $h_{t}^{b}$ in both directions. The final output is the concatenate of the hidden states in both directions.

(7) $$h_{t}=[h_{t}^{f},h_{t}^{b}]$$

BiLSTM layers usually have more parameters and are susceptible to over-fitting. We perform a dropout operation on the feature matrix of the BiLSTM output to reduce the risk of over-fitting. Finally, we perform the flatten operation on the feature matrix and then concatenate it with the output of the Global Feature Channel.

#### 4.2.2. Global Feature Channel

**Figure 12:**
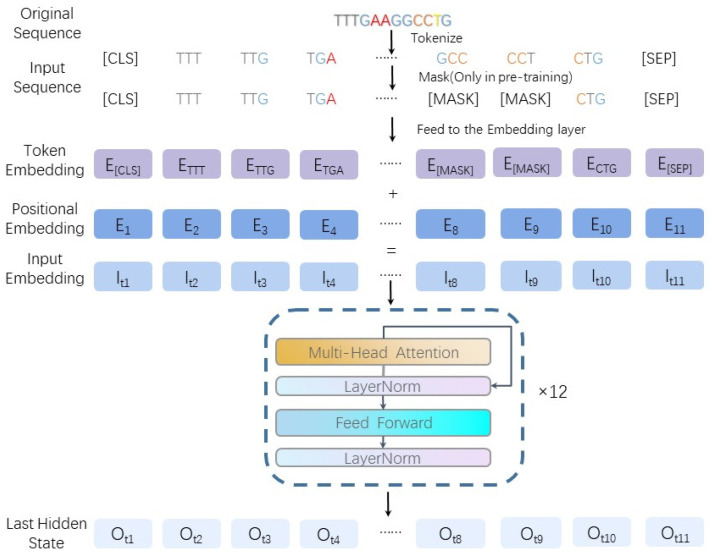
The architecture of DNABert.

DNABERT uses k-mer to cut the original DNA segment into short sequences of nucleobases of length k for input in the next step. This helps to connect the nucleobases to the ones that follow them, combining richer contextual information. The model architecture of DNABERT [28,29] is shown in Figure 12.

### 4.3. Network Architecture of DeepDualEnhancer-Genomic

On the new dataset we collected (six cell lines), we attempted to incorporate genomic signals. This is because we believe that combining the DNA sequence and genomic signals can better accomplish the enhancer recognition task. We still use the model architecture designed in Section 4.2. The architecture of the Global Feature Channel part is retained, while genomic signals are added.

**Figure 13:**
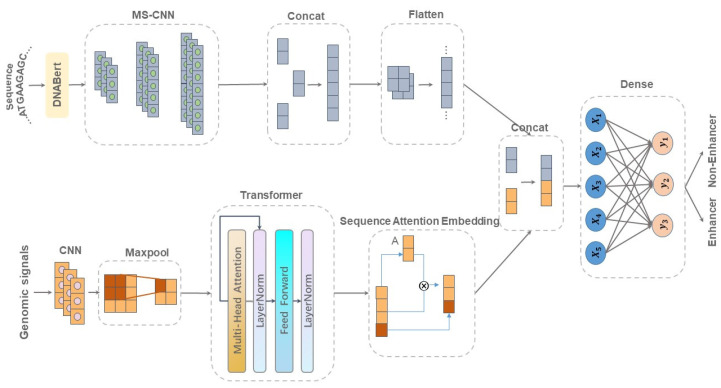
Network architecture of DeepDualEnhancer-genomic.

Firstly, shallow features are still extracted by convolution and pooling, and the feature dimensions are decreased to reduce the amount of post-computation. Then, the input genomic signals are converted into a representation in the high dimensional space by the encoder in the transformer, which captures the semantic and contextual information of the input sequence. The encoder contains a multi-head attention mechanism, a feed-forward neural network, and layer normalization. Finally, we obtain the final feature matrix by using the sequence attention method. In sequence attention, we first calculated the sequence weights through two layers of attention. Then, we performed a weighted sequence embedding representation and enhanced the index of the predicted enhancer region. Because genomic signals are more of a macroscopic concept, the variation in different genomic signals can only be observed on longer DNA sequences. Therefore, we selected the genomic signals with a length of 1,000,000 bp and centered on the enhancer fragment to input. In the final sequence attention approach, the features were assigned weights, and the features of the enhancer segments were concatenated with them. After the final concatenation with the sequence features, the final prediction results were output through the fully connected layer. The network architecture is shown in Figure 13.

In the subsequent results section, we have demonstrated through multiple experiments that inputting genomic signals is helpful in improving the performance of the method on independent testing datasets.

### 4.4. Model Training

The models in this study were trained on a GeForce RTX 4090, with the batch size parameter set to 32 and the Adam optimizer used. The best-performing model was obtained by minimizing the loss function BCEWithLogitsLoss. BCEWithLogitsLoss is used for predicting whether DNA sequence segments are enhancers due to its suitability for binary classification and numerical stability. This loss function performs effective gradient propagation during training and allows sample weighting. To prevent over-fitting, multiple dropout functions were added to the model, and the validation set was set to 10%. In addition to saving the final trained model weights, we also saved the model weights that performed best on the validation set during the training process. The specific training parameters are shown in Table 8.

The formulas for the metrics in the Section 2 are shown below:

(8) $$SN=\frac{TP}{TP+FN}$$

(9) $$SP=\frac{TN}{TN+FP}$$

(10) $$ACC=\frac{TP+TN}{TP+FP+TN+FN}$$

(11) $$MCC=\frac{TP\times TN-FP\times FN}{\sqrt{\left(TP+FN\right)\times \left(TP+FP\right)\times \left(TN+FN\right)\times \left(TN+FP\right)}}$$

TP (True Positive): the predicted value is 1 and the true value is 1, i.e., true positive; FP (False Positive): the predicted value is 1 and the true value is 0, i.e., false positive; TN (True Negative): the predicted value is 0 and the true value is 0, i.e., true negative; FN (False Negative): the predicted value is 0 and the true value is 1, i.e., false negative.

## 5. Conclusions

In this study, we propose a new framework, DeepDualEnhancer, based on deep learning, which accurately predicts enhancers using DNA sequence information. DeepDualEnhancer has good robustness and a good generalization ability. Unlike previous enhancer prediction methods, DeepDualEnhancer adopts a dual-channel network architecture and utilizes two different encoding methods, including fine-tuned DNABert. At the same time, BiLSTM and MS-CNN networks were used to extract the shallow and deep features contained in the sequence, which better completed the prediction task. In the predictions of Stage 1 and Stage 2, the best performance of multiple metrics was achieved on the independent test set. In the first stage, the scores were 0.8200 (ACC), 0.6455 (MCC), and 0.8662 (AUC), respectively. The second-stage scores were 0.9150 (ACC), 0.8423 (MCC), and 0.9864 (AUC). We also collected large-scale datasets of six different cell lines. The DeepDualEnhancer-genomic network architecture was designed, along with a sequence attention embedding method. We simultaneously input DNA sequence information and genomic/epigenetic signals to predict enhancer segments. Through experimental verification, it has been proven that inputting genomic signals can improve the accuracy of prediction.

## Figures and Tables

**Table 1:** Results of cross-validation (Stage 1).

First Stage	SN	SP	ACC	MCC	AUC
iEnhancer-PsedeKNC	0.7731	0.7630	0.7678	0.5400	0.8500
iEnhancer-5Step	0.8110	0.8350	0.8230	0.6500	-
DeployEnhancer	0.7325	0.7642	0.7483	0.4980	0.7694
EnhancerP-2L	0.9077	0.9259	0.9168	0.8340	0.9400
iEnhancer-CNN	0.7588	0.8888	0.8063	0.6929	0.8957
Enhancer-BERT	0.7950	0.7300	0.7620	0.5250	-
iEnhancer-KL	0.8322	0.8524	0.8423	0.6800	-
iEnhancer-RF	0.7364	0.7871	0.7618	0.5264	0.8400
piEnPred	0.9228	0.8047	0.8788	0.7660	0.9603
iEnhancer-RD	0.9100	0.7650	0.7880	0.5760	0.8440
DeepSTARR	0.5678	0.8533	0.7123	0.4425	0.8057
Enhancer-LSTMAtt	0.7304	0.8006	0.7655	0.5339	0.8259
DeepDualEnhancer	0.8511	0.8989	0.8750	0.7508	0.9364

**Table 2:** Results of cross-validation (Stage 2).

Second Stage	SN	SP	ACC	MCC	AUC
iEnhancer-PsedeKNC	0.6262	0.6441	0.6341	0.2700	0.6900
iEnhancer-5Step	0.7530	0.6080	0.6810	0.3700	-
DeployEnhancer	0.7965	0.3828	0.5896	0.1970	0.6068
EnhancerP-2L	0.6221	0.6182	0.6193	0.2400	0.9000
iEnhancer-CNN	0.7364	0.7680	0.7643	0.4505	0.8109
iEnhancer-KL	0.9340	0.9287	0.9313	0.8600	-
iEnhancer-RF	0.6846	0.5661	0.6253	0.2529	0.6700
piEnPred	0.6554	0.7094	0.6824	0.3654	0.7568
iEnhancer-RD	0.8400	0.5700	0.7050	0.4260	0.7920
DeepSTARR	0.3914	0.7712	0.5815	0.1806	0.6141
Enhancer-LSTMAtt	0.6765	0.6024	0.6395	0.2804	0.6439
DeepDualEnhancer	0.6715	0.6334	0.6523	0.3087	0.6885

**Table 3:** Comparison with state-of-the-art methods (Stage 1).

First Stage	SN	SP	ACC	MCC	AUC
iEnhancer-2L	0.7100	0.7500	0.7300	0.4604	0.8062
EnhancerPred	0.7350	0.7450	0.7400	0.4800	0.8013
iEnhancer-EL	0.7100	0.7850	0.7475	0.4964	0.8173
iEnhancer-5Step	0.8200	0.7600	0.7900	0.5800	-
DeployEnhancer	0.7550	0.7600	0.7550	0.5100	0.7704
iEnhancer-ECNN	0.7520	0.7850	0.7690	0.5370	0.8320
EnhancerP-2L	0.7810	0.8105	0.7950	0.5907	-
iEnhancer-CNN	0.7825	0.7900	0.7750	0.5850	-
iEnhancer-XG	0.7400	0.7750	0.7575	0.5150	-
Enhancer-DRRNN	0.7330	0.8010	0.7670	0.5350	0.8370
Enhancer-BERT	0.8000	0.7120	0.7560	0.5140	-
iEnhancer-RF	0.7850	0.8100	0.7975	0.5952	0.8600
spEnhancer	0.8300	0.7150	0.7725	0.5793	0.8235
iEnhancer-EBLSTM	0.7550	0.7950	0.7720	0.5340	0.8350
iEnhancer-GAN	0.8110	0.7580	0.7840	0.5670	-
piEnPred	0.8250	0.7840	0.8040	0.6099	-
iEnhancer-RD	0.8100	0.7650	0.7880	0.5760	0.8440
iEnhancer-MFGBDT	0.7679	0.7955	0.7750	0.5607	-
DeepSTARR	0.6000	0.9200	0.7600	0.5489	0.8506
Enhancer-LSTMAtt	0.7950	0.8150	0.8050	0.6101	0.8588
iEnhancer-DCSV	0.8025	0.7765	0.7895	0.5809	0.8527
DeepDualEnhancer	0.7555	0.8850	0.8200	0.6455	0.8662

**Table 4:** Comparison with state-of-the-art methods (Stage 2).

Second Stage	SN	SP	ACC	MCC	AUC
iEnhancer-2L	0.4700	0.7400	0.6050	0.2181	0.6678
EnhancerPred	0.4500	0.6500	0.5500	0.1020	0.5790
iEnhancer-EL	0.5400	0.6800	0.6100	0.2222	0.6801
iEnhancer-5Step	0.7400	0.5300	0.6350	0.2800	-
DeployEnhancer	0.8315	0.4561	0.6849	0.3120	0.6714
ES-ARCNN	0.8600	0.4500	0.6560	0.3399	-
iEnhancer-ECNN	0.7910	0.5640	0.6780	0.3680	0.7480
EnhancerP-2L	0.6829	0.7922	0.7250	0.4624	-
iEnhancer-CNN	0.6525	0.7610	0.7500	0.3232	-
iEnhancer-XG	0.7000	0.5700	0.6350	0.2720	-
Enhancer-DRRNN	0.8580	0.8400	0.8490	0.6990	-
iEnhancer-RF	0.9300	0.7700	0.8500	0.7091	0.9700
spEnhancer	0.9100	0.3300	0.6200	0.3703	0.6253
iEnhancer-EBLSTM	0.8120	0.5360	0.6580	0.3240	0.6880
iEnhancer-GAN	0.9610	0.5370	0.7490	0.5050	-
piEnPred	0.7000	0.7500	0.7250	0.4506	-
iEnhancer-RD	0.8400	0.5700	0.7050	0.4260	0.7920
iEnhancer-MFGBDT	0.7255	0.6681	0.6850	0.3862	-
DeepSTARR	0.5600	0.8700	0.7150	0.4523	0.8013
Enhancer-LSTMAtt	0.9900	0.8000	0.8950	0.8047	0.9637
iEnhancer-DCSV	0.9910	0.6230	0.8070	0.6609	0.8686
DeepDualEnhancer	1.000	0.8300	0.9150	0.8423	0.9864

**Table 5:** Training dataset.

Task	Positive Sample	Negative Sample
Stage I	1484	1484
Stage II	742	742

**Table 6:** Independent dataset.

Task	Positive Sample	Negative Sample
Stage I	200	100
Stage II	100	100

**Table 7:** BENGI Dataset.

Cell Line	Positive Sample	Negative Sample
GM12878+HeLa-S3	9421	28,263
HMEC	687	2061
IMR90	568	1704
K562	706	2118
NHEK	588	1764

**Table 8:** Training parameters.

Parameter	Value Setting
Embedding dim	32
Learning rate	0.0001
Batch size	32
Data shuffle	True
Epoch	20

## Data Availability

The DeepDualEnhancer project’s source code and datasets are publicly available at https://github.com/UPC020317/DeepDualEnhancer (accessed on 22 October 2024).
